# Molecular Analysis of Ciprofloxacin Resistance Mechanisms in Malaysian ESBL-Producing *Klebsiella pneumoniae* Isolates and Development of Mismatch Amplification Mutation Assays (MAMA) for Rapid Detection of *gyrA* and *parC* Mutations

**DOI:** 10.1155/2014/601630

**Published:** 2014-04-10

**Authors:** Farah Al-Marzooq, Mohd Yasim Mohd Yusof, Sun Tee Tay

**Affiliations:** Department of Medical Microbiology, Faculty of Medicine, University of Malaya, 50603 Kuala Lumpur, Malaysia

## Abstract

Ninety-three Malaysian extended-spectrum *β*-lactamase (ESBL)-producing *Klebsiella pneumoniae* isolates were investigated for ciprofloxacin resistance. Two mismatch amplification mutation (MAMA) assays were developed and used to facilitate rapid detection of *gyrA* and *parC* mutations. The isolates were also screened for plasmid-mediated quinolone resistance (PMQR) genes including *aac(6*′*)-Ib-cr, qepA*, and *qnr*. Ciprofloxacin resistance (MICs 4– ≥ 32 **μ**g/mL) was noted in 34 (37%) isolates, of which 33 isolates had multiple mutations either in *gyrA* alone (*n* = 1) or in both *gyrA* and *parC* regions (*n* = 32). *aac(6*′*)-Ib-cr* was the most common PMQR gene detected in this study (*n* = 61), followed by *qnrB* and *qnrS* (*n* = 55 and 1, resp.). Low-level ciprofloxacin resistance (MICs 1-2 **μ**g/mL) was noted in 40 (43%) isolates carrying *qnrB* accompanied by either *aac(6*′*)-Ib-cr *(*n* = 34) or a single *gyrA* 83 mutation (*n* = 6). Ciprofloxacin resistance was significantly associated with the presence of multiple mutations in *gyrA* and *parC* regions. While the isolates harbouring *gyrA* and/or *parC* alteration were distributed into 11 PFGE clusters, no specific clusters were associated with isolates carrying PMQR genes. The high prevalence of ciprofloxacin resistance amongst the Malaysian ESBL-producing *K. pneumoniae* isolates suggests the need for more effective infection control measures to limit the spread of these resistant organisms in the hospital.

## 1. Introduction

The emergence and spread of extended-spectrum *β*-lactamase (ESBL)-producing organisms have posed a great challenge to clinicians worldwide. As ESBL-producing organisms are usually resistant to many other antimicrobial agents, limited therapeutic options are available for treatment of infections caused by these organisms [1]. Ciprofloxacin is one of the therapeutic choices for infections caused by bacteria belonging to the family* Enterobacteriaceae*. This antibiotic acts by inhibiting bacterial DNA gyrase and topoisomerase IV which are required for replication [2].

Resistance to fluoroquinolones (FQ) is now common in many ESBL-producing Gram-negative bacteria including* Klebsiella pneumoniae* [3, 4]. FQ resistance has been linked to specific amino acid substitutions in the chromosomal quinolone resistance determining regions (QRDRs) in GyrA and B subunits of DNA gyrase and ParC and E subunits of topoisomerase IV [5]. Mutations at Ser83 and Asp87 codons of GyrA subunit and Ser80 and Glu84 codons of ParC subunit have been commonly reported in FQ resistant* K. pneumoniae* isolates worldwide [6–8]. DNA sequencing is the gold standard method for the detection of these mutations; however, this method is expensive, laborious, and time consuming. Hence, cheaper, simpler, and rapid methods are required to facilitate mutation detection. A few assays have been developed for rapid detection of mutations in* gyrA* and/or* parC* genes of* Campylobacter jejuni* [9],* Escherichia coli* [10], and* Neisseria gonorrhoeae* [11] using mismatch amplification mutation assay (MAMA), a modified polymerase chain reaction that permits discriminatory amplification of specific allele sequences at QRDRs [12].

Plasmid-mediated quinolone resistance (PMQR) genes, including* qnr, aac(6*′*)-Ib-cr* and efflux pumps, are known to confer low-level FQ resistance [13]. Quinolone target protection by Qnr proteins are widely distributed in* Enterobacteriaceae* worldwide [14]. Until now, six Qnr families, namely, Qnr A, B, C, D, S, and VC, have been identified (http://www.lahey.org/qnrStudies/). While* qnrA, B,* and *S* genes are commonly detected at variable rates in* K. pneumoniae* worldwide [3, 13, 15],* qnrC* and *D* have been reported at low rates amongst* K. pneumoniae* isolates in China [16]. Moreover, a variant of aminoglycoside acetyltransferase (AAC(6′)-Ib-cr) with the ability to modify and inactivate ciprofloxacin has been widely spread in* K. pneumoniae* isolates from Asia [17, 18] and worldwide [3, 14].

FQ resistance may also arise as a result of reduced intracellular drug accumulation caused by porin loss or active efflux pump [5]. QepA, a quinolone-specific efflux pump, has been identified in* Escherichia coli* isolates from several Asian countries such as Japan, Korea, and China [19–21] but was rarely detected in* K. pneumoniae* [22, 23].

There is a paucity of data on the prevalence and the genetic determinants associated with ciprofloxacin resistance in Malaysian* K. pneumoniae* isolates. Hence, this study was conducted to identify chromosomal as well as plasmid-mediated mechanisms of ciprofloxacin resistance in a group of Malaysian ESBL-producing* K. pneumoniae* isolates. To facilitate rapid detection of* gyrA* and* parC* mutations, two mismatch amplification mutation assays (MAMA-PCR) were developed and validated in this study.

## 2. Materials and Methods

### 2.1. Bacterial Isolates

A group of 93 nonduplicate ESBL-producing* K. pneumoniae* isolates from patients attending to University of Malaya Medical Centre and a private hospital in Kuala Lumpur, Malaysia, in 2010–2012 were investigated in this study. The isolates were confirmed as* K. pneumoniae* using a PCR assay targeting the internal transcribed spacer unit of the bacteria [24]. Confirmation of ESBL production was performed using Cefpodoxime Combination Disc Kit (Oxoid, UK).

### 2.2. Antibiotic Susceptibility Testing

Minimum inhibitory concentration (MIC) of ciprofloxacin was determined by *E*-test strips (bioMéreiux, Marcy L'Etoile, France) on Mueller-Hinton agar (Oxoid, UK) in accordance with the Clinical and Laboratory Standards Institute (CLSI) guidelines [25]. MIC values ≥4, 2, and ≤1 *μ*g/mL were used to define resistance, intermediate susceptibility, and susceptibility to ciprofloxacin, respectively.

### 2.3. Development of MAMA-PCR for* gyrA* and* parC* Mutations Detection

Two duplex PCR assays* (gyrA*83 +* parC*80 assay and* gyrA*87 +* parC*84 assay) were developed for the simultaneous detection of mutations in Ser83 codon of GyrA subunit and Ser80 codon of ParC subunit, and Asp87 codon of GyrA subunit and Glu84 codon of ParC subunit, respectively.

**Figure 1:**
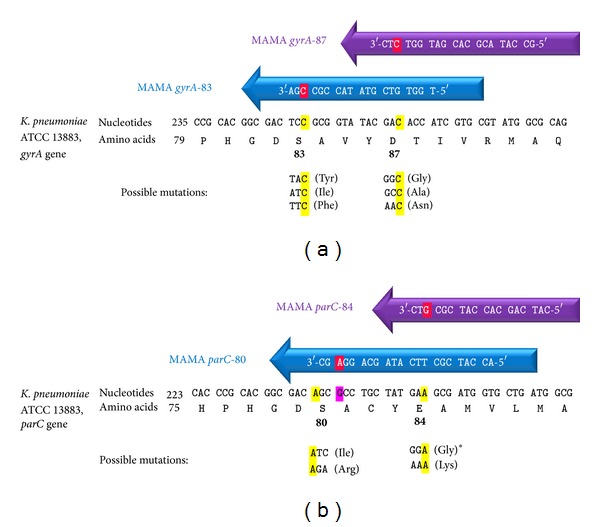
MAMA-PCR primers for* gyrA* (a) and* parC* (b) mutation detection. Red highlighted nucleotides are the mismatched nucleotides at the 3′ end of each MAMA primer. Mismatches were positioned at the conserved nucleotides of each codon (highlighted by yellow) located at the 3rd nucleotide from the 3′ end of each primer, except for* parC*80 primer where the conserved nucleotide (1st nucleotide in the* parC*80 codon) was excluded from the MAMA primer and the alteration was situated at a nucleotide outside the coding region (pink highlighted nucleotide). Quality control strains with the expected mutations shown in the figure were used for the assay development and optimization except the mutation with ∗ which was not available.

Universal* gyrA* and* parC* forward primers [16] were used together with the reverse primers (MAMA primers) designed in this study for the amplification of* gyrA* (Ser83 and Asp87) and* parC* (Ser80 and Glu84) genetic regions (Figure 1). MAMA primer design was performed using NCBI/Primer-BLAST tool (http://www.ncbi.nlm.nih.gov/tools/primer-blast/). The reverse primers were complementary to the wild-type alleles of* gyrA* and* parC* sequences of* K. pneumoniae* strain ATCC 13883 (GenBank accession numbers: DQ673325 and AF303641, resp.), except for a mismatch at the antepenultimate (−3) nucleotide of the 3′ end of each MAMA primer, which was included to improve allele discrimination. The MAMA primer:template mismatches included in this study were C:C (in* gyrA*83 and 87), A:G (in* parC*80), G:A (in* parC*84). The selection of the mismatches was based on previous observations of their effects on the overall PCR yield [26]. The presence of a single primer:template mismatch has minimal effect on the PCR yield; thus the wild-type gene can be amplified efficiently. In case of mutation(s), PCR efficiency will be extremely reduced due to the presence of additional mismatch(es) at the 3′ end of the MAMA primer which will not bind to the template; thus, amplification of the target gene is failed [27].

The performance of the primers was first evaluated using monoplex PCR prior to use in the duplex PCR assays which were finally optimized for the simultaneous detection of mutations in Ser83 codon of GyrA subunit with Ser80 codon of ParC subunit and in Asp87 codon of GyrA subunit with Glu84 codon of ParC subunit.

For the first duplex PCR assay, the concentrations of primers were optimized to 0.4 *μ*M for each* parC*80 MAMA primer and* parC* universal forward primer, in addition to 0.25 *μ*M for each* gyrA*83 MAMA primer and* gyrA* universal forward primer. For the second assay, the concentrations of primers were optimized to 0.45 *μ*M for each* parC*84 MAMA primer and* parC* universal forward primer, in addition to 0.2 *μ*M for each of* gyrA*87 MAMA primer and* gyrA* universal forward primer. The primer mixtures were added to a final PCR reaction volume of 20 *μ*L containing 4 *μ*L of 5x HOT FIREPol Blend Master Mix (Solis BioDyne, Estonia) which was comprised of 200 *μ*M of each dNTP, 0.05 U/*μ*L of DNA polymerase, and 1.5 mM MgCl_2_. Finally, 1 *μ*L (<100 ng) of boiled bacterial extract [28] was added to each reaction. Amplification was carried out on a Veriti 96-well thermal cycler (Applied Biosystems, USA) programmed as follows: initial denaturation at 95°C for 10 min, followed by 30 cycles of denaturation at 95°C for 30 s, annealing at 56°C for 40 s and extension at 72°C for 50 s, and a final extension step at 72°C for 7 min. PCR products were analysed on a 2% agarose gel prestained with 0.5 *μ*g/mL ethidium bromide in 0.5x TBE buffer.

*K. pneumoniae* strains with known mutations in* gyrA* (45 isolates) and* parC* (10 isolates) were used as quality control strains for the assay optimization and validation [29].

### 2.4. Detection of* gyrA* and* parC* Mutations by MAMA-PCR

Following validation of the MAMA-PCR assays, the 93 clinical isolates investigated in this study were tested for alterations in* gyrA* and/or* parC* regions. For confirmation purpose, amplification and sequence analysis of the entire coding regions of* gyrA* and* parC* for 25 randomly selected isolates were performed as described previously [16]. The nucleotide sequences and deduced proteins were analyzed by NCBI tools and BioEdit software (version 7) and were compared with those of GyrA and ParC subunits of* K. pneumoniae* strain ATCC 13883 (GenBank accession numbers: DQ673325 and AF303641, resp.).

### 2.5. Detection of Plasmid-Mediated Quinolone Resistance (PMQR) Genes

All isolates were subjected to screening using a multiplex PCR assay for the detection of* qnr* types (*A, B, C,* and* S*) [13] and a monoplex PCR assay for the detection of* qnrD* type [30]. Detection of efflux pump (*qepA*) and the aminoglycoside acetyltransferase (*aac(6*′*)-Ib*) was performed using a multiplex PCR assay [13]. Allele-specific PCR assay was used to identify the* cr *mutation in* aac(6*′*)-Ib* [31]. To confirm the PCR results, representative amplicons of each PMQR gene were sequenced. Additionally, nine isolates from different susceptibility categories (resistant, intermediately susceptible, and susceptible to ciprofloxacin) were selected for sequence determination of the entire* qnrB* gene, as described previously [32]. The nucleotide sequences and deduced proteins were compared to the reference sequences in Lahey website (http://www.lahey.org/qnrStudies/) and GenBank database using BLAST search engine (http://blast.ncbi.nlm.nih.gov/Blast.cgi).

### 2.6. Pulsed-Field Gel Electrophoresis (PFGE)

PFGE was used to determine the genetic relationship of the isolates as described previously [33]. Fragments generated by restriction with XbaI enzyme (New England Biolabs, USA) were separated by the CHEF-DR II system (Bio-Rad Laboratories, USA). The resultant banding patterns were analysed with BioNumerics software, version 7.1 (Applied Maths, Belgium), by the unweighted pair group method with arithmetic mean (UPGMA) algorithm. Cluster designation was based on isolates showing ≥80% relatedness.

### 2.7. Statistical Analyses

Categorical variables were compared by the Chi-square or Fisher's exact test and continuous variables were compared by Mann-Whitney *U* test. The relationship between ciprofloxacin MIC values with the number of* gyrA* and/or* parC* mutations and with the total number of quinolone resistance determinants was assessed by calculating Spearman's correlation coefficient. The total number of quinolone resistance determinants was calculated by adding the number of PMQR genes and the number of mutations in Ser83 and/or Asp87 codons of GyrA subunit and in Ser80 and/or Glu84 codons of ParC subunit. All tests were two-tailed and a *P* value < 0.05 was considered statistically significant. All statistical analyses were performed by PASW software version 18 (SPSS, Chicago, IL, USA).

## 3. Results

Reduced susceptibility to ciprofloxacin (resistance and intermediate susceptibility) was observed in 66 (71%) isolates investigated in this study. Ciprofloxacin MIC values of the isolates ranged from 0.032 to ≥32 *μ*g/mL with MIC_90_ and MIC_50_ equal to ≥32 and 2 *μ*g/mL, respectively. Based on ciprofloxacin MICs, the 93 ESBL-producing* K. pneumoniae* isolates were grouped into three susceptibility categories. The MIC values with quinolone resistance determinants in each category are shown in Table 1.

### 3.1. Development and Validation of MAMA-PCR

**Figure 2:**
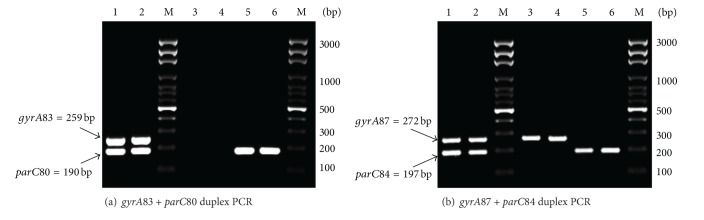
Agarose gel electrophoresis image of PCR products generated from duplex MAMA-PCR assays (a)* gyrA*83 +* parC*80 and (b)* gyrA*87 +* parC*84. Identification of each target was based on the expected product size. Lanes 1-2 represent PCR products generated in the presence of the wild-type alleles. Lanes 3–6 are examples of products generated in case of mutations in one gene or in both target genes. M: DNA molecular size marker (100 bp DNA Ladder, Solis BioDyne, Estonia).

The universal forward and MAMA reverse primers generated a PCR product from the wild-type gene in the absence of mutation(s). On the other hand, PCR was inhibited in the presence of mutation(s) (two or more mismatches at the 3′ end of the MAMA primer); therefore, negative PCR result was an indication of mutation in the corresponding genetic region. MAMA primers were able to distinguish wild types from mutations for all of the quality control strains (45 isolates for* gyrA* and 10 isolates for* parC*). The four MAMA monoplex PCR assays were then combined into two duplex assays (*gyrA*83 +* parC*80 assay and* gyrA*87 +* parC*84 assay).  Figure 2 shows the results of MAMA duplex assays for some of the isolates investigated in this study.

### 3.2. Detection of* gyrA* and* parC* Mutations by MAMA-PCR Assays

Alterations in* gyrA* and/or* parC* genetic regions were detected in 41 of the 93* K. pneumoniae* clinical isolates by MAMA method as shown in Table 2. Mutations in* gyrA* were detected in 44% (*n* = 41) of the isolates, of which 17 isolates had both Ser83 and Asp87 alterations, and 24 isolates had Ser83 mutation detected either alone in ciprofloxacin susceptible (*n* = 5) or intermediately susceptible isolates (*n* = 3) or coupled with* parC* mutations in ciprofloxacin resistant isolates (*n* = 16).

Alterations in ParC subunit of DNA topoisomerase IV were detected in 34.4% (*n* = 32) of the isolates, of which 30 isolates had Ser80 mutation and two isolates had Glu84 alteration. Mutations in* parC* were detected in ciprofloxacin resistant isolates which had single or multiple* gyrA* mutations.

MAMA findings were confirmed by sequence analysis of the entire coding regions of* gyrA* and* parC* for selected isolates (seven isolates with the wild type of* gyrA* and* parC* and 18 isolates with MAMA results indicative of* gyrA* and/or* parC* alterations). Detected amino acid substitutions in GyrA were Ser83Ile (*n* = 10), Ser83Tyr (*n* = 4), and Ser83Phe + Asp87Ala (*n* = 4), whilst substitutions in ParC were Ser80Ile (*n* = 15), Ser80Arg (*n* = 1), and Glu84Lys (*n* = 2) (Table 2).

### 3.3. PMQR Genes

*aac(6′)-Ib* gene was detected in 74 (79.6%) of the isolates, of which 61 (65.6%) carried the* cr* variant.* qnr* genes were detected from 56 isolates (60.2%), of which 55 carried* qnrB* (59.1%) and one carried* qnrS* (1.1%). Sequence analysis of* qnrB* gene in nine randomly selected isolates revealed 100% nucleotide sequence identity with* qnrB1* (*n* = 4),* qnrB6* (*n* = 3), and* qnrB7* (*n* = 2) (GenBank accession numbers DQ351241, GQ914054, and EU043311, resp.). The deduced proteins (223 amino acids) also exhibited 100% amino acid identity with QnrB1, QnrB6, and QnrB7 (GenBank accession numbers DQ351241, ADH03417, and ABW03156, resp.).

*aac(6′)-Ib-cr* and* qnr* genes were detected from isolates which were susceptible (17 and 11 isolates, resp.), intermediately susceptible (29 and 32 isolates, resp.), and resistant to ciprofloxacin (15 and 13 isolates, resp.). Interestingly, 47 isolates (50.2%) harbored both* qnrB* and* aac(6*′*)-Ib-cr* genes; thus the association between both genes was statistically significant (*P* < 0.001). Neither* qepA* efflux pump nor* qnrA, C,* and *D* genes were detected in this study.

### 3.4. The Relationship between Ciprofloxacin MIC and FQ Resistance Determinants

Table 1 shows the increase in the ciprofloxacin MICs of our isolates which was accompanied by a stepwise accumulation of FQ resistance determinants. The increase in ciprofloxacin MICs is correlated strongly with the increase in the total number of FQ resistance determinants (both* gyrA* and/or* parC* mutations and PMQR genes) (Spearman's correlation coefficient = 0.918; *P* < 0.001).

The lowest MIC values were noted in the isolates lacking any FQ resistance determinants (0.032–0.047 *μ*g/mL). For the isolates with one FQ resistance determinant, MICs of isolates expressing* aac(6*′*)-Ib-cr* alone were significantly lower (0.094–0.38 *μ*g/mL) than those of isolates expressing* qnr* gene alone or having a single* gyrA*83 mutation (0.5–0.75 *μ*g/mL) (*P* = 0.001). MICs of isolates expressing two FQ resistance determinants (*qnr* gene accompanied by either* aac(6*′*)-Ib-cr* or a single* gyrA*83 mutation) were significantly higher (1-2 *μ*g/mL) compared to those of isolates expressing one of the above mentioned genes alone (*P* < 0.001).

Of note, 34 out of 40 isolates which demonstrated reduced susceptibility to ciprofloxacin (MIC = 1-2 *μ*g/mL) harbored both* qnrB* and* aac(6*′*)-Ib-cr* genes; thus both genes were significantly associated with low-level ciprofloxacin resistance (*P* < 0.001).

There is significant association between the resistance phenotype and the presence of more than one mutation in* gyrA* and/or* parC* (*P* < 0.001), as most (33 out of 34) ciprofloxacin resistant isolates harbored 2-3 mutations in* gyrA* and/or* parC* codons. These isolates demonstrated significantly higher MIC values (4–≥32 *μ*g/mL) compared to those of isolates harboring single* gyrA*83 mutation with and without* qnrB* gene (MICs 0.5–2 *μ*g/mL, *P* < 0.001) and those harboring* qnr* and/or* aac(6*′*)-Ib-cr* genes without any alterations in QRDRs (MICs 0.094–2 *μ*g/mL, *P* < 0.001). Thus, the increase in ciprofloxacin MICs is correlated strongly with the increase in the total number of mutations in* gyrA* and/or* parC* subunits (Spearman's correlation coefficient = 0.78; *P* < 0.001).

Notably,* aac(6*′*)-Ib-cr* and* qnrB* were detected in some of the ciprofloxacin resistant isolates (15 and 13 isolates, resp.). The real contribution of PMQR on ciprofloxacin MIC is not clear in the ciprofloxacin resistant isolates as there is nonsignificant difference in ciprofloxacin MICs of the resistant isolates with and without* aac(6*′*)-Ib-cr* and* qnrB* genes (*P* > 0.05).

### 3.5. PFGE

**Figure 3:**
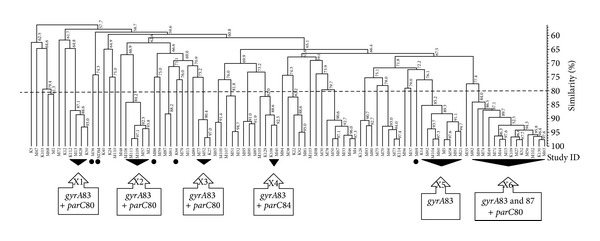
PFGE dendrogram of the 93* K. pneumoniae* isolates investigated in this study. Alterations in GyrA and/or ParC subunits were detected in the isolates which belong to 11 clusters. Black triangles represent clusters with multiple isolates possessing the same* gyrA* and/or* parC* mutations. Black circles represent monoisolate clusters with* gyrA* and/or* parC* mutations. The dashed line represents the similarity level (80%) used in the clusters definition.

The 93 isolates investigated in this study were differentiated into 41 PFGE clusters (Figure 3). The isolates harboring* gyrA* and/or* parC* alteration were distributed into 11 clusters, of which six clusters (X1–X6) were composed of 2–14 genetically related isolates, whereas the remaining five clusters were comprised of only one isolate each. Identical* gyrA* and/or* parC* mutations were found amongst isolates within the same cluster, with the only exception of two ciprofloxacin resistant isolates in cluster X4. In this cluster, two highly related isolates (92.3%) harboring* gyrA*83 and* parC*84 mutations were genetically related (less than 89%) to another two isolates without any* gyrA* and* parC* mutations (one was sensitive and the other was intermediately susceptible to ciprofloxacin). While* gyrA* and/or* parC* alteration were limited to isolates within 11 clusters, PMQR genes were detected in isolates distributed into 38 clusters. The FQ resistance determinants of the isolates in different clusters were presented in the supplementary data file (Supplementary Material available online at http://dx.doi.org/10.1155/2014/601630).

## 4. Discussion

A big proportion of our isolates (71%) were nonsusceptible (resistant and intermediately susceptible) to ciprofloxacin, which is a common finding in ESBL-producing isolates as reported in several countries such as Taiwan (59.1%) [34], France (60.3%) [3], and UK (62.3%) [35]. According to the latest study of antimicrobial resistance trends (SMART) in the Asia-Pacific region, ciprofloxacin nonsusceptibility in* K. pneumoniae* was much higher in the ESBL-producing isolates (65.8%) compared to the non-ESBL-producing isolates (7.4%) [36]. This may explain the reason why our ciprofloxacin nonsusceptibility rate (71%) was higher than the rate reported in a previous Malaysian study (18%) because the isolates investigated in that study were a mixture of ESBL and non-ESBL-producing* K. pneumoniae* isolates [37].

In this study, two multiplex MAMA-PCR assays have been successfully developed for the detection of mutations in* gyrA*83 +* parC*80 and in* gyrA*87 +* parC*84 codons of quinolone resistance determining regions in* K. pneumoniae*. To the best of our knowledge, no specific assay has been developed previously for the detection of alterations in the QRDRs of* K. pneumoniae.* A few monoplex PCR assays using MAMA method have been developed to detect alterations in bacterial QRDRs. For* C. jejuni* and* N. gonorrhoeae*, MAMA primers were designed to amplify particular mutations in the* gyrA* codon and not the wild type [9, 11]. On the contrary, the primers used in the* E. coli* MAMA-PCR method were designed in a way that* gyrA* or* parC* wild types were amplified and no PCR product would be obtained if mutations were present [10]. A similar approach was used in designing the MAMA primers in this study. Additionally, instead of using four monoplex MAMA-PCR assays as described in the* E. coli* study, two duplex assays were designed for simultaneous amplification of the* gyrA* and* parC* genetic regions. The duplex assay strategy is expected to facilitate rapid detection of mutations in the* gyrA* and* parC* since it shortens the time and the steps involved. The MAMA method developed in this study is rapid and cost effective compared to DNA sequencing approach and is useful for screening of a large number of* K. pneumoniae* isolates. Isolates with results suggestive of mutations can be selected for sequence analysis in order to define the amino acid at the mutation site.

DNA sequence analysis for selected isolates revealed several types of amino acid substitutions in GyrA (Ser83Ile, Ser83Tyr, Ser83Phe, and Asp87Ala) and ParC (Ser80Ile, Ser80Arg, and Glu84Lys). Similar amino acid substitutions were detected in GyrA regions of* K. pneumoniae* isolates from Malaysia [29] and other Asian countries [16, 38]. No data is available on ParC alterations in the Malaysian* K. pneumoniae* isolates; however, the amino acid substitutions detected in our study (Ser80Ile, Ser80Arg, and Glu84Lys) have been reported previously in* K. pneumoniae* isolates from other Asian countries such as Singapore [39], Japan [6], and Taiwan [38].

In agreement with previous reports [6, 40], multiple alterations in GyrA and/or ParC have been associated with ciprofloxacin resistance. Isolates with single alterations in* gyrA*83 exhibited reduced susceptibility to ciprofloxacin (MIC = 0.5–2 *μ*g/mL). This observation has been reported previously [5] and is considered as the first step for the development of full resistance to ciprofloxacin. Ciprofloxacin MICs of our isolates increased with the acquisition of additional mutations in* gyrA* and/or* parC* genetic regions. This was expected as previous studies have shown that the progression from FQ susceptible towards resistant phenotype is a gradual process, starting from mutations in* gyrA*, the primary target of FQ, and followed by* parC* alterations which has a complementary role in the development of higher resistance [2, 41].

Surprisingly, we had one ciprofloxacin resistant isolate (MIC ≥ 32 *μ*g/mL) which lacked any mutation in* gyrA* and* parC* genes but harbored both* qnrB* and* aac(6*′*)-Ib-cr* genes. Similar observations have been reported in isolates from other geographical regions [39, 42, 43]. The possible involvement of resistance mechanisms such as the reduction of bacterial drug uptake due to active efflux system (for instance, OqxAB) and/or membrane impermeability due to porin loss, or mutation in other genetic regions such as* gyrB* or* parE*, is yet to be explored.

The high prevalence of PMQR genes (65.6% for* aac(6*′*)-Ib-cr* and 60.2% for* qnr*) in our ESBL-producing* K. pneumoniae* isolates is in agreement with previous findings from other parts of the world [34, 44]. This high prevalence can be attributed to the coexistence of ESBL and PMQR genes on the same plasmid, as reported previously [34, 45]. Similarly, the simultaneous detection of both* qnrB* and* aac(6*′*)-Ib-cr* genes in 50.2% of the isolates is an indication that they are most likely located on the same plasmid, as shown in previous studies [17, 45].

*aac(6′)-Ib-cr* was the most common PMQR gene detected in this study. No information is available on the prevalence of this gene in the Malaysian* Enterobacteriaceae* isolates; however, studies from other Asian countries such as China [18], Korea [17], and Thailand [44] confirmed the emergence and spread of this gene amongst* K. pneumoniae* isolates.

*qnrB* was the predominant* qnr* gene identified in this study, in agreement with a previous report from our hospital [29] and reports from other Asian countries [15, 46].* qnrB1, qnrB6*, and* qnrB7* were detected by sequence analysis in some of our isolates. Both* qnrB1* and* qnrB6* have been previously detected in* K. pneumoniae* isolates from Malaysia [29] and other parts of Southeast Asia [38, 44], whereas* qnrB7* has only been reported in two* K. pneumoniae* isolates from Norway and Sweden [47].

*qnrS* was detected at a very low frequency in our isolates, in contrast to a recent Thai report whereby this gene was the dominant* qnr* type in* K. pneumoniae* isolates [44]. Other* qnr* types including* qnrA, C,* and *D* were not detected in this study. The prevalence of* qnr* genes is variable from time to time and in different geographical locations [13]. This phenomenon has been attributed to the variations in the* qnr*-carrying plasmids which can also possess multiple antibiotic resistance genes. As a result of antibiotic selective pressure, some plasmids may dominate in certain clinical settings [48].* qepA* efflux pump gene was not detected in our isolates. This was expected as the prevalence of this gene is generally low worldwide [22, 23].

In this study, the progressive increase in ciprofloxacin MICs of our isolates is correlated with the stepwise accumulation of FQ resistance determinants. Isolates with single FQ resistance determinant (a single PMQR gene (*qnr* or* aac(6*′*)-Ib-cr*) or a single* gyrA*83 mutation) demonstrated ciprofloxacin MICs (0.094–0.75 *μ*g/mL) lower than those of isolates expressing two FQ resistance determinants (1-2 *μ*g/mL) including two PMQR genes (*qnrB* and* aac(6*′*)-Ib-cr*) or* qnrB* with a single* gyrA*83 mutation (Table 1). Our results indicate that the effect of different FQ resistance determinants on ciprofloxacin MIC is cumulative, as reported previously [14].

Although the expression of* qnr* and* aac(6*′*)-Ib-cr* confers low-level ciprofloxacin resistance, it may have a negative impact on the therapeutic efficacy of ciprofloxacin as observed in rat animal models of experimental infection with* qnr* producing* K. pneumoniae* [49, 50]. Moreover, the expression of these genes can increase the mutant prevention concentration, which is the lowest antimicrobial concentration required to prevent the emergence of resistant mutants; thus, resistant mutants can be selected under ciprofloxacin therapeutic levels [51, 52].

The PFGE results in this study show the evidence of the spread of ciprofloxacin resistant isolates harboring GyrA and/or ParC alterations by clonal expansion, as identical mutations in* gyrA* and/or* parC* were detected from genetically related isolates. Similar findings have also been reported previously by other investigators [35]. Interestingly, both* gyrA*83 and* parC*84 mutations were detected in two highly related isolates (92.3%), which were genetically related to another two isolates without any* gyrA* and* parC* mutations. All the four isolates were hospital-associated (data not shown); therefore, it is possible that they have originated from a common ancestor in the hospital environment. Ciprofloxacin resistance in two of the four isolates was probably caused by* de novo* mutations in* gyrA*83 and* parC*84 induced by ciprofloxacin selective pressure, as previously observed in* E. coli* [53]. This assumption was supported by the finding of both* qnrB* and* aac(6*′*)-Ib-cr* in the two ciprofloxacin resistant isolates as both genes have the ability to enhance the selection of chromosomal mutations [2].

PMQR genes were widely distributed into isolates within different PFGE clusters. Horizontal dissemination of the plasmids carrying PMQR genes is probably responsible for the high prevalence of PMQR genes in our isolates due to their wide distribution amongst genetically unrelated isolates [44].

## 5. Conclusions

A high prevalence of ciprofloxacin resistance was reported amongst the Malaysian ESBL-producing* K. pneumoniae* isolates investigated in this study. The current scenario can complicate the clinical management of patients because very few antimicrobial agents are active against these bacteria. This study also identified chromosomal and plasmid-mediated genetic determinants associated with ciprofloxacin resistance. The MAMA method developed in this study is simple, cost effective, and rapid for detection of* gyrA* and* parC* mutations. It is important for epidemiologic investigations particularly when a large number of bacterial isolates need to be screened. The high prevalence of PMQR genes in our isolates is alarming as these genes can be widely spread via plasmids, confer low-level ciprofloxacin resistance, and facilitate the selection of chromosomal mutations implicated in higher level ciprofloxacin resistance. Regular surveillance of ciprofloxacin resistance determinants and molecular epidemiologic investigations are essential in order to follow up the progress of resistance development and spread in our hospital settings.

## Supplementary Material

The distribution of 93 *K. pneumoniae* isolates into 41 PFGE clusters. The ciprofloxacin MIC and detection of plasmid-mediated quinolone resistance (PMQR) genes and chromosomal mutations in *gyrA* and/or *parC* gene regions for each isolate are shown.

## Figures and Tables

**Table 1:** Ciprofloxacin susceptibility patterns and fluoroquinolone (FQ) resistance determinants detected in the 93 *K*. *pneumoniae* isolates investigated in this study.

Ciprofloxacin susceptibility	MIC (*μ*g/mL)	Number of isolates	FQ resistance determinants		Total number of *gyrA* and/or *parC* alterations	Total number of FQ resistance determinants
			PMQR* genes (*n*)	*gyrA* and/or *parC* alterations (*n*)		
Susceptible (*n* = 27, 29%)	0.032–0.047	2	None (2)	None (2)	None	None
	0.094–0.38	12	*aac(6*′*)-Ib-cr* (12)	None (12)	None	1
	0.5–0.75	5	*qnrB *(2), *qnrS* (1) None (2)	None (3) *gyrA*83 (2)	None 1	1 1
	1	8	*qnrB* + *aac(6*′*)-Ib-cr* (5) *qnrB* (3)	None (5) *gyrA*83 (3)	None 1	2 2

Intermediately susceptible (*n* = 32, 34%)	2	32	*qnrB* + *aac(6*′*)-Ib-cr* (29) *qnrB* (3)	None (29) *gyrA*83 (3)	None 1	2 2

Resistant (*n* = 34, 37%)	4–6	4	None (4)	*gyrA*83 + *parC*80 (4)	2	2
	≥32	30	*qnrB* + *aac(6*′*)-Ib-cr* (8) *qnrB* + *aac(6*′*)-Ib-cr* (2) *qnrB* + *aac(6*′*)-Ib-cr* (1) *qnrB* + *aac(6*′*)-Ib-cr* (1), *aac(6*′*)-Ib-cr* (2) *qnrB* + *aac(6*′*)-Ib-cr* (1)	*gyrA*83 + *parC*80 (10) *gyrA*83 + *parC*84 (2) *gyrA*83 + *gyrA*87 (1) *gyrA*83 + *gyrA*87 + *parC*80 (16) None (1)	2 2 2 3 None	2 or 4 4 4 3–5 2

*PMQR: Plasmid-mediated quinolone resistance.

**Table 2:** Alterations in *gyrA* and *parC* genes detected by MAMA-PCR and confirmed by sequence analysis for selected isolates.

Total number of mutations	Number of isolates	Alterations detected by MAMA-PCR				Confirmation by sequencing (*n*)	
		*gyrA *		*parC *			
		83	87	80	84	*gyrA *	*parC *
None	52	None	None	None	None	Wild type (7)	Wild type (7)
1	8	Mutation	None	None	None	Ser83Tyr (4)	ND
2	1	Mutation	Mutation	None	None	ND	ND
	14	Mutation	None	Mutation	None	Ser83Ile (8)	Ser80Ile (9), Ser80Arg (1)
	2	Mutation	None	None	Mutation	Ser83Ile (2)	Glu84Lys (2)
3	16	Mutation	Mutation	Mutation	None	Ser83Phe + Asp87Ala (4)	Ser80Ile (6)

ND: not done.
