# Supplementary material for: Molecular Analysis of Ciprofloxacin Resistance Mechanisms in Malaysian ESBL-Producing Klebsiella pneumoniae Isolates and Development of Mismatch Amplification Mutation Assays (MAMA) for Rapid Detection of gyrA and parC Mutations
Source: Biomed Res Int. 2014 Apr 10;2014:601630. doi: 10.1155/2014/601630 (PMC4000930; doi:10.1155/2014/601630)
Supplement: Supplementary file 1 — The distribution of 93 K. pneumoniae isolates into 41 PFGE clusters. The ciprofloxacin MIC and detection of plasmid-mediated quinolone resistance (PMQR) genes and chromosomal mutations in gyrA and/or parC gene regions for each isolate are shown. [file 601630.f1.pdf]

**ID**	**PFGE group**	**PMQR genes**		**MAMA PCR results**		
				gyrA		***pa[illegible]***
		qnr	***aac(6′)-Ib-cr***	**83**	**87**	**80**
K52	A1	-VE	-VE	**mutation**	**mutation**	**mutation**
K109	A1	-VE	-VE	**mutation**	**mutation**	**mutation**
K130	A1	-VE	-VE	**mutation**	**mutation**	**mutation**
M15	A1	-VE	-VE	**mutation**	**mutation**	**mutation**
M27	A1	-VE	-VE	**mutation**	**mutation**	**mutation**
M44	A1	-VE	-VE	**mutation**	**mutation**	**mutation**
M49	A1	-VE	-VE	**mutation**	**mutation**	**mutation**
M56	A1	-VE	-VE	**mutation**	**mutation**	**mutation**
M63	A1	-VE	-VE	**mutation**	**mutation**	**mutation**
M71	A1	-VE	-VE	**mutation**	**mutation**	**mutation**
M74	A1	-VE	-VE	**mutation**	**mutation**	**mutation**
M82	A1	-VE	Positive	**mutation**	**mutation**	**mutation**
M99	A1	-VE	Positive	**mutation**	**mutation**	**mutation**
M102	A1	-VE	-VE	**mutation**	**mutation**	**mutation**
M35	A2	*qnrB*	Positive	**Wild type**	**Wild type**	**Wild type**
K14	A3	*qnrB* (*qnrB7*)*	-VE	**mutation**	**Wild type**	**Wild type**
M5	A3	-VE	-VE	**mutation**	**Wild type**	**Wild type**
M21	A3	-VE	-VE	**mutation**	**Wild type**	**Wild type**
M58	A3	*qnrB*	-VE	**mutation**	**Wild type**	**Wild type**
M60	A3	*qnrB* (*qnrB7*)	-VE	**mutation**	**Wild type**	**Wild type**
M91	A3	*qnrB*	-VE	**mutation**	**Wild type**	**Wild type**
M92	A3	*qnrB*	-VE	**mutation**	**Wild type**	**Wild type**
M104	A3	*qnrB*	-VE	**mutation**	**Wild type**	**Wild type**
M69	A4	-VE	-VE	**mutation**	**mutation**	**mutation**
M37	A5	*qnrB*	Positive	**Wild type**	**Wild type**	**Wild type**
M96	A6	*qnrB*	Positive	**Wild type**	**Wild type**	**Wild type**
K114	A7	*qnrB*	Positive	**Wild type**	**Wild type**	**Wild type**
M66	A7	*qnrB*	Positive	**Wild type**	**Wild type**	**Wild type**
M70	A7	*qnrB*	Positive	**Wild type**	**Wild type**	**Wild type**
M73	A7	*qnrB*	Positive	**Wild type**	**Wild type**	**Wild type**
M75	A8	*qnrB*	Positive	**Wild type**	**Wild type**	**Wild type**
K126	A9	-VE	Positive	**Wild type**	**Wild type**	**Wild type**
M80	A9	-VE	Positive	**Wild type**	**Wild type**	**Wild type**
M93	A9	-VE	Positive	**Wild type**	**Wild type**	**Wild type**
M4	A10	*qnrB*	Positive	**Wild type**	**Wild type**	**Wild type**
M33	A10	*qnrB*	Positive	**Wild type**	**Wild type**	**Wild type**
M46	A10	*qnrB*	Positive	**Wild type**	**Wild type**	**Wild type**
M50	A10	*qnrB*	Positive	**Wild type**	**Wild type**	**Wild type**
M67	A10	*qnrB*	Positive	**Wild type**	**Wild type**	**Wild type**
M78	A10	*qnrB*	Positive	**Wild type**	**Wild type**	**Wild type**

M30	A11	*qnrB*	Positive	**Wild type**	**Wild type**	**Wild type**
M98	A12	-VE	Positive	**Wild type**	**Wild type**	**Wild type**
M103	A13	-VE	Positive	**Wild type**	**Wild type**	**Wild type**
K22	A14	-VE	Positive	**Wild type**	**Wild type**	**Wild type**
K92	A14	-VE	-VE	**Wild type**	**Wild type**	**Wild type**
M38	A14	*qnrB* (*qnrB1*)	Positive	**Wild type**	**Wild type**	**Wild type**
M81	A14	-VE	-VE	**Wild type**	**Wild type**	**Wild type**
M84	A15	-VE	Positive	**Wild type**	**Wild type**	**Wild type**
K106	A16	*qnrB* (*qnrB6*)	Positive	**mutation**	**Wild type**	**Wild type**
M40	A16	*qnrB*	Positive	**mutation**	**Wild type**	**Wild type**
K129	A16	-VE	Positive	**Wild type**	**Wild type**	**Wild type**
M105	A16	*qnrB*	Positive	**Wild type**	**Wild type**	**Wild type**
M85	A17	*qnrB*	Positive	**Wild type**	**Wild type**	**Wild type**
M90	A17	*qnrB*	Positive	**Wild type**	**Wild type**	**Wild type**
M95	A17	*qnrB*	Positive	**Wild type**	**Wild type**	**Wild type**
M32	A18	-VE	Positive	**Wild type**	**Wild type**	**Wild type**
M51	A18	*qnrB*	Positive	**Wild type**	**Wild type**	**Wild type**
M107	A18	*qnrB*	Positive	**Wild type**	**Wild type**	**Wild type**
M65	A19	*qnrB*	Positive	**Wild type**	**Wild type**	**Wild type**
M100	A19	*qnrB*	Positive	**Wild type**	**Wild type**	**Wild type**
K27	A20	*qnrB* (*qnrB1*)	Positive	**mutation**	**Wild type**	**mutation**
M52	A20	*qnrB*	Positive	**mutation**	**Wild type**	**mutation**
M77	A20	*qnrB*	Positive	**mutation**	**Wild type**	**mutation**
M89	A21	*qnrB*	Positive	**Wild type**	**Wild type**	**Wild type**
M11	A22	-VE	Positive	**Wild type**	**Wild type**	**Wild type**
M79	A23	*qnrB* (*qnrB6*)	Positive	**Wild type**	**Wild type**	**Wild type**
K66	A24	-VE	-VE	**mutation**	**Wild type**	**mutation**
M61	A25	*qnrB* (*qnrB1*)	-VE	**Wild type**	**Wild type**	**Wild type**
M87	A25	-VE	Positive	**Wild type**	**Wild type**	**Wild type**
M59	A26	*qnrB*	Positive	**Wild type**	**Wild type**	**Wild type**
M86	A27	-VE	-VE	**mutation**	**Wild type**	**mutation**
M2	A28	*qnrB*	Positive	**mutation**	**Wild type**	**mutation**
M57	A28	*qnrB*	Positive	**mutation**	**Wild type**	**mutation**
M106	A28	*qnrB*	Positive	**mutation**	**Wild type**	**mutation**
M109	A28	*qnrB*	Positive	**mutation**	**Wild type**	**mutation**
M111	A28	*qnrB*	Positive	**mutation**	**Wild type**	**mutation**
M48	A29	*qnrB*	Positive	**Wild type**	**Wild type**	**Wild type**
M110	B1	*qnrB*	-VE	**Wild type**	**Wild type**	**Wild type**
K24	B2	*qnrB* (*qnrB6*)	Positive	**Wild type**	**Wild type**	**Wild type**
K40	B3	*qnrB*	Positive	**Wild type**	**Wild type**	**Wild type**
NDM	C1	*qnrB*	Positive	**mutation**	**mutation**	**mutation**
M36	C2	*qnrB*	Positive	**mutation**	**mutation**	**Wild type**
K50	D1	-VE	-VE	**mutation**	**Wild type**	**mutation**

K112	D1	-VE	-VE	**mutation**	**Wild type**	**mutation**
M13	D1	-VE	-VE	**mutation**	**Wild type**	**mutation**
M28	D1	-VE	-VE	**mutation**	**Wild type**	**mutation**
K12	D2	*qnrS*	-VE	**Wild type**	**Wild type**	**Wild type**
M72	D3	*qnrB*	Positive	**Wild type**	**Wild type**	**Wild type**
M1	E1	*qnrB*	Positive	**Wild type**	**Wild type**	**Wild type**
M68	E1	*qnrB*	Positive	**Wild type**	**Wild type**	**Wild type**
K103	E2	-VE	Positive	**Wild type**	**Wild type**	**Wild type**
M47	E3	*qnrB*	Positive	**Wild type**	**Wild type**	**Wild type**
K5	E4	*qnrB* (*qnrB1*)	Positive	**Wild type**	**Wild type**	**Wild type**

* *qnrB* alleles determined by sequencing

rC	Sequencing		Total number of mutations	Total number of FQ resistance determinants	Ciprofl
84	*gyrA*	*parC*			MIC (µg/ml)
**Wild type**	not done	not done	3	3	≥32
**Wild type**	S83F+ D87A	S80I	3	3	≥32
**Wild type**	S83F+ D87A	S80I	3	3	≥32
**Wild type**	S83F+ D87A	not done	3	3	≥32
**Wild type**	S83F+ D87A	not done	3	3	≥32
**Wild type**	not done	S80I	3	3	≥32
**Wild type**	not done	not done	3	3	≥32
**Wild type**	not done	not done	3	3	≥32
**Wild type**	not done	not done	3	3	≥32
**Wild type**	not done	not done	3	3	≥32
**Wild type**	not done	S80I	3	3	≥32
**Wild type**	not done	S80I	3	4	≥32
**Wild type**	not done	not done	3	4	≥32
**Wild type**	not done	not done	3	3	≥32
**Wild type**	not done	not done	0	2	2
**Wild type**	S83Y	not done	1	2	1
**Wild type**	S83Y	not done	1	1	0.5
**Wild type**	not done	not done	1	1	0.5
**Wild type**	S83Y	not done	1	2	1
**Wild type**	S83Y	not done	1	2	2
**Wild type**	not done	not done	1	2	2
**Wild type**	not done	not done	1	2	1
**Wild type**	not done	not done	1	2	2
**Wild type**	not done	not done	3	3	≥32
**Wild type**	not done	not done	0	2	1
**Wild type**	**Wild type**	**Wild type**	0	2	2
**Wild type**	not done	not done	0	2	2
**Wild type**	not done	not done	0	2	2
**Wild type**	not done	not done	0	2	2
**Wild type**	not done	not done	0	2	2
**Wild type**	not done	not done	0	2	1
**Wild type**	not done	not done	0	1	0.19
**Wild type**	not done	not done	0	1	0.094
**Wild type**	not done	not done	0	1	0.125
**Wild type**	not done	not done	0	2	2
**Wild type**	not done	not done	0	2	1
**Wild type**	not done	not done	0	2	2
**Wild type**	not done	not done	0	2	2
**Wild type**	not done	not done	0	2	2
**Wild type**	**Wild type**	**Wild type**	0	2	2

**Wild type**	not done	not done	0	2	2
**Wild type**	not done	not done	0	1	0.094
**Wild type**	not done	not done	0	1	0.25
**Wild type**	not done	not done	0	1	0.125
**Wild type**	not done	not done	0	0	0.047
**Wild type**	not done	not done	0	2	1
**Wild type**	not done	not done	0	0	0.032
**Wild type**	not done	not done	0	1	0.38
**mutation**	S83I	E84K	2	4	≥32
**mutation**	S83I	E84K	2	4	≥32
**Wild type**	**Wild type**	**Wild type**	0	1	0.19
**Wild type**	**Wild type**	**Wild type**	0	2	2
**Wild type**	not done	not done	0	2	2
**Wild type**	not done	not done	0	2	2
**Wild type**	not done	not done	0	2	2
**Wild type**	not done	not done	0	1	0.38
**Wild type**	not done	not done	0	2	2
**Wild type**	not done	not done	0	2	2
**Wild type**	not done	not done	0	2	2
**Wild type**	not done	not done	0	2	2
**Wild type**	S83I	S80I	2	4	≥32
**Wild type**	S83I	not done	2	4	≥32
**Wild type**	not done	S80I	2	4	≥32
**Wild type**	not done	not done	0	2	2
**Wild type**	not done	not done	0	1	0.094
**Wild type**	**Wild type**	**Wild type**	0	2	≥32
**Wild type**	S83I	S80R	2	2	≥32
**Wild type**	not done	not done	0	1	0.75
**Wild type**	not done	not done	0	1	0.125
**Wild type**	not done	not done	0	2	2
**Wild type**	not done	S80I	2	2	≥32
**Wild type**	S83I	S80I	2	4	≥32
**Wild type**	not done	not done	2	4	≥32
**Wild type**	not done	S80I	2	4	≥32
**Wild type**	not done	not done	2	4	≥32
**Wild type**	not done	not done	2	4	≥32
**Wild type**	not done	not done	0	2	2
**Wild type**	not done	not done	0	1	0.5
**Wild type**	**Wild type**	not done	0	2	2
**Wild type**	not done	**Wild type**	0	2	2
**Wild type**	not done	S80I	3	5	≥32
**Wild type**	not done	not done	2	4	≥32
**Wild type**	S83I	S80I	2	2	4

**Wild type**	S83I	S80I	2	2	4
**Wild type**	S83I	S80I	2	2	6
**Wild type**	S83I	S80I	2	2	6
**Wild type**	not done	not done	0	1	0.5
**Wild type**	not done	not done	0	2	1
**Wild type**	not done	not done	0	2	2
**Wild type**	not done	not done	0	2	2
**Wild type**	not done	not done	0	1	0.094
**Wild type**	not done	not done	0	2	2
**Wild type**	**Wild type**	**Wild type**	0	2	2

oxacin
**phenotype**
**R**
**R**
**R**
**R**
**R**
**R**
**R**
**R**
**R**
**R**
**R**
**R**
**R**
**R**
I
S
S
S
S
I
I
S
I
**R**
S
I
I
I
I
I
S
S
S
S
I
S
I
I
I
I

I
S
S
S
S
S
S
S
**R**
**R**
S
I
I
I
I
S
I
I
I
I
**R**
**R**
**R**
I
S
**R**
**R**
S
S
I
**R**
**R**
**R**
**R**
**R**
**R**
I
S
I
I
**R**
**R**
**R**

**R**
**R**
**R**
S
S
I
I
S
I
I
